# Safety and Acceptability of Day-Care Surgery Versus Short-Stay Surgery in Patients Undergoing Laparoscopic Totally Extraperitoneal (TEP) Inguinal Hernia Repair

**DOI:** 10.7759/cureus.94862

**Published:** 2025-10-18

**Authors:** Rahul Kumar Keshari, Manzoor Ahmad, Imad Ali, Wasif Mohammad Ali, Ahmad Sadiq, Saddam Hussain, Md Shahzada Alam

**Affiliations:** 1 General Surgery, Jawaharlal Nehru Medical College and Hospital, Aligarh Muslim University, Aligarh, IND

**Keywords:** day-care surgery, inguinal hernia, laparoscopic tep, mesh repair, short-stay surgery

## Abstract

Introduction: Inguinal hernia repair is the most common general surgical procedure worldwide. Laparoscopic totally extraperitoneal (TEP) repair offers the advantages of reduced pain, faster recovery, and early return to activity. With increasing focus on enhanced recovery and cost-effectiveness, day-care surgery is being explored as an alternative to short-stay admission. This study evaluated the safety, efficacy, and acceptability of day-care versus short-stay TEP inguinal hernia repair.

Methods: A prospective randomized study was conducted at Jawaharlal Nehru Medical College, Aligarh, from April 2023 to March 2025. Forty-one patients with uncomplicated symptomatic inguinal hernia were enrolled: 16 underwent day-care surgery (Group A), and 25 underwent short-stay surgery (Group B). All patients underwent laparoscopic TEP repair under general anesthesia. Demographics, hernia characteristics, operative time, complications, postoperative pain, ambulation, return to work, and patient satisfaction were assessed.

Results: Both groups were comparable in age, body mass index (BMI), American Society of Anesthesiologists (ASA) Grade, and operative duration. Right-sided and lateral hernias predominated, with larger hernias in Group A (p = 0.0028). Mean operative time was similar (66.38 ± 4.60 vs. 66.56 ± 4.06 minutes; p = 0.8933). Postoperative pain, nausea, and vomiting were mild and comparable. Complications were rare, limited to urinary retention and one vascular injury in Group B. No infection, seroma, scrotal edema, or recurrence occurred. Day-care patients resumed work earlier and reported higher satisfaction.

Conclusion: Day-care laparoscopic TEP repair is safe, effective, and well accepted, offering faster recovery, improved satisfaction, and better resource utilization, compared with short-stay surgery.

## Introduction

Inguinal hernia repair is the most common general surgical procedure performed worldwide, with approximately 20 million surgeries conducted annually. Open or laparoscopic mesh repair remains the recommended treatment for symptomatic inguinal hernias [1]. Laparoscopic repair has emerged as a feasible alternative to open repair [2], gaining popularity due to the emphasis on enhanced recovery protocols [3]. Compared to open surgery, laparoscopic repair is associated with significantly less postoperative pain and an earlier return to work when performed by experienced surgeons [4,5].

The advent of laparoscopy revolutionized inguinal hernia management. The first case of minimally invasive inguinal hernia repair was reported in 1992, followed by the introduction of the totally extraperitoneal (TEP) repair by McKernan and Laws in 1993 [6]. Multiple studies have shown that laparoscopic hernia repair reduces postoperative pain, decreases the need for analgesics, and allows an earlier return to normal activity compared with conventional open repair [7].

Advancements in surgical techniques, understanding of disease processes, and anesthetic management have improved patient outcomes and reduced hospital stay. With adequate recovery, defined as pain-free ambulation, normal oral intake, and self-care, patients can be discharged safely in the immediate postoperative period. This concept gave rise to day-care surgery. Dr. Nicoll is regarded as the pioneer of modern ambulatory surgery, having reported his 10-year experience with 7,392 pediatric cases, including hernias and cleft lip and palate, at Glasgow Paediatric Hospital [8]. In day-care surgery, patients are admitted, operated, and discharged on the same day after adequate recovery.

Day-care surgery is increasingly accepted worldwide due to its advantages: reduced inpatient bed occupancy, lower psychological stress by minimizing disruption from familiar surroundings, decreased risk of nosocomial infections, higher patient satisfaction, and cost-effectiveness for both patients and healthcare systems. These benefits are particularly relevant in resource-constrained settings like India, which bears 20% of the global disease burden but has only 6% of hospital beds worldwide. India has fewer than half as many hospital beds per 1,000 population compared to countries such as China and Brazil and less than 35% of the global average [7].

This study aimed to evaluate whether laparoscopic TEP repair can be safely, feasibly, and acceptably performed as a day-care procedure.

## Materials and methods

Patients and methods

This prospective randomized study was conducted in the Department of Surgery, Jawaharlal Nehru Medical College, Aligarh, between April 2023 and March 2025, after obtaining Institutional Ethics Committee approval. A total of 41 patients with inguinal hernia were enrolled, of which 16 were randomly assigned to Group A for day-care surgery and the remaining 25 were assigned to Group B for short-stay surgery, using a simple random sampling technique.

Inclusion and exclusion criteria

The study included patients aged above 14 years with American Society of Anesthesiologists (ASA) Physical Status I-II, who provided informed consent and had access to essential home facilities such as toilet, transport, and telephone. Consecutive patients presenting with uncomplicated, symptomatic unilateral or bilateral inguinal hernia were considered eligible. Patients were excluded if they resided more than one hour away from the hospital, lacked an adult caretaker for the first 24 hours after discharge, or belonged to ASA Grade III or higher. Additional exclusion criteria were uncontrolled diabetes, alcohol abuse, chronic obstructive pulmonary disease (COPD), severe asthma, marked dyspnea, epilepsy, pregnancy, coagulation disorders, recurrent hernia, multiple prior abdominal surgeries, chronic analgesic use, alcohol or substance dependence, or the need for concomitant surgical procedures. Patients unwilling to provide consent were also excluded.

**Figure 1:**
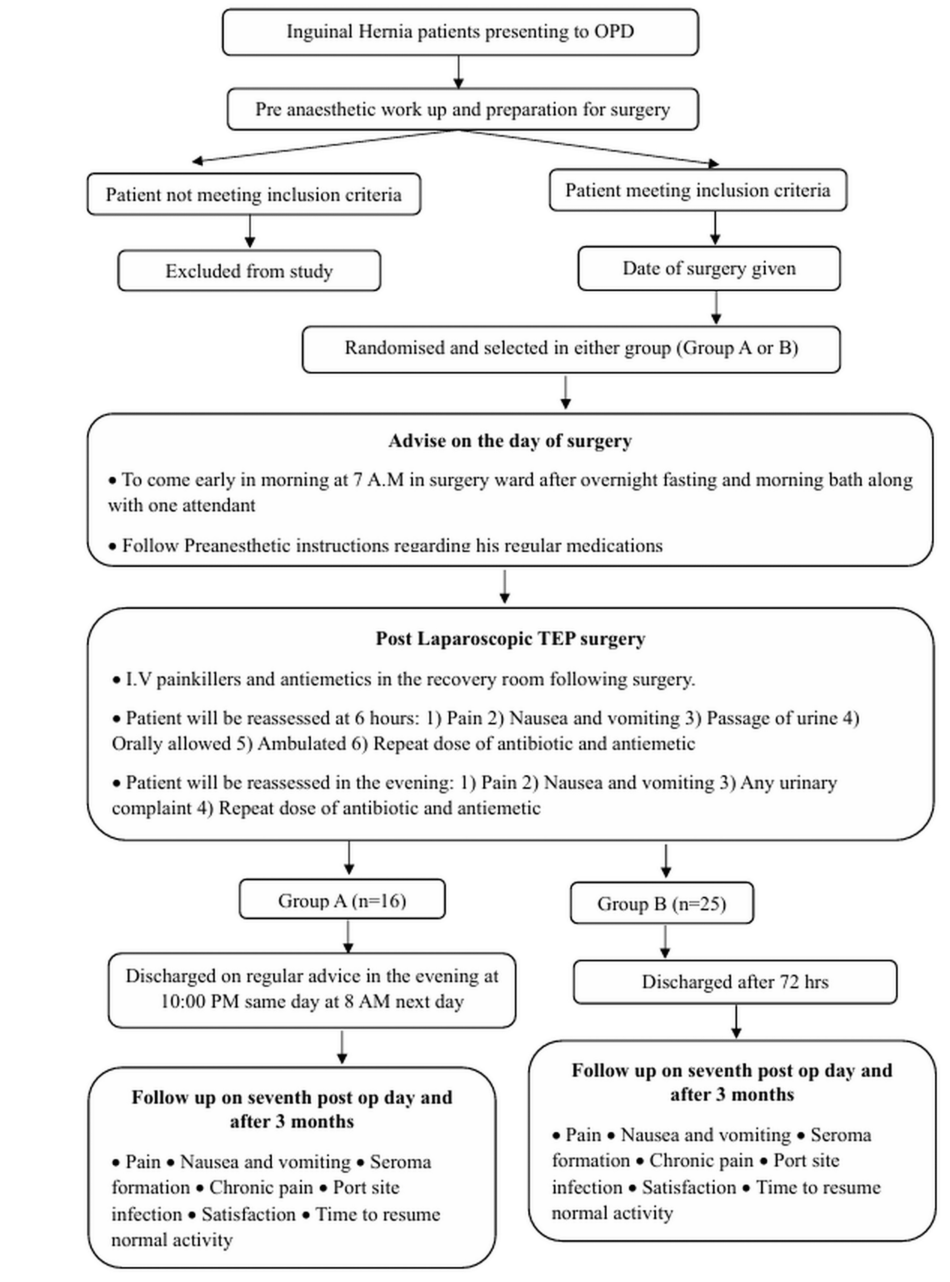
Recruitment and study process timeline

Surgical technique

All patients underwent laparoscopic TEP inguinal hernia repair under general anesthesia following routine pre-anesthetic evaluation and overnight fasting. Patients were admitted with an attendant, given prophylactic antibiotics, and positioned supine with slight Trendelenburg tilt. A 10-12 mm infra-umbilical incision was used to access the rectus sheath, and a balloon trocar created the extraperitoneal space. Two additional 5 mm ports were inserted under direct visualization. Standard preperitoneal dissection was carried out to identify key landmarks including Cooper’s ligament, inferior epigastric vessels, and Bogros’ space, with careful reduction of hernia sacs. A 10 × 15 cm polypropylene/polyester mesh was placed to cover the myopectineal orifice and selectively fixed at Cooper’s ligament and rectus muscle to minimize nerve injury and chronic pain [9,10].

Postoperatively, patients received IV analgesics and antiemetics with reassessment for pain, nausea, urinary complaints, oral intake, and ambulation. Day-care patients (Group A) were discharged the same evening with written and verbal instructions, medications, and emergency contacts, while short-stay patients (Group B) were observed for 72 hours before discharge. All patients were reviewed at seven days and at three months, with additional telephonic follow-up, for assessment of pain, seroma, infection, chronic discomfort, return to activity, and overall satisfaction.

Data and statistical analysis

Data were prospectively collected using a structured case record form. Demographic details, comorbidities, type of hernia, operative duration, and intraoperative events were recorded. Postoperative parameters including pain scores, nausea/vomiting, urinary complaints, ambulation, oral intake, and need for rescue medication were assessed at six hours and on the evening of the surgery. Discharge timing, hospital stay, and follow-up at seven days and at three months were documented for complications, return to activity, and patient satisfaction, with interim events captured telephonically

Data were entered in Microsoft Excel (Microsoft Corp., Redmond, WA, USA) and analyzed using IBM SPSS Statistics for Windows, Version 27.0 (Released 2019; IBM Corp., Armonk, NY, USA) and GraphPad Prism Version 5 (Dotmatics, Boston, MA, USA). Continuous variables were expressed as mean and standard deviation, while categorical variables were presented as counts and percentages. Between-group comparisons were performed using the independent t-test for continuous variables, paired t-test where applicable, and chi-square or Fisher’s exact test for categorical variables. A p-value of less than 0.05 was considered statistically significant.

## Results

A total of 41 patients were included in the study, with 16 in Group A (day-care) and 25 in Group B (short-stay). All 16 (100%) patients in Group A and 25 (100%) in Group B were male. The mean age was 33.75 ± 11.21 years in Group A and 39.32 ± 10.58 years in Group B, with no statistically significant difference (p = 0.1163). The mean body mass index (BMI) was comparable between the groups (23.87 ± 2.55 vs. 24.36 ± 3.23; p = 0.6157). The mean ASA grade was 1.00 ± 0.00 in Group A and 1.04 ± 0.20 in Group B, showing no significant difference (p = 0.4307).

Right inguinal hernia was the predominant presentation observed in 15 patients (93.8%) in Group A and 23 (92.0%) in Group B, while left inguinal hernia occurred in 1 (6.3%)in Group A and 2 (8.0%) in Group B (p = 0.8337, chi-square value: 0.0441, df = 1). The most common type of hernia was lateral (14 (87.5%) in Group A, 20 (80.0%) in Group B), followed by medial hernia (1 (6.3%) in Group A, 2 (8.0%) in Group B), and femoral hernia (1 (6.3%) in Group A, 3 (12.0%) in Group B). Differences were not statistically significant (p = 0.8035, chi-square value: 0.4376, df = 2) (Table 1).

**Table 1:** Types and sides of hernia in the study population Chi-square tests were used to compare variables.

Parameter	Group A (day-care, n = 16)	Group B (short-stay, n = 25)	p-value
Hernia side	Right: 93.8%	Right: 92.0%	0.8337
	Left: 6.3%	Left: 8.0%	
Hernia type	Lateral: 87.5%	Lateral: 80.0%	0.8035
	Medial: 6.3%	Medial: 8.0%	
	Femoral: 6.3%	Femoral: 12.0%	

The mean hernia size however was significantly larger in Group A (2.69 ± 0.48) compared to Group B (1.84 ± 0.98; p = 0.0028).

All patients underwent laparoscopic TEP repair. The mean operative duration was similar between the groups (66.38 ± 4.60 minutes in Group A vs. 66.56 ± 4.06 minutes in Group B; p = 0.8933).

Postoperative outcomes were favorable in both groups. The mean VAS pain score at six hours was 2.75 ± 0.86 in Group A and 3.04 ± 0.84 in Group B (p = 0.2913) and was 1.19 ± 0.40 and 1.20 ± 0.41, respectively, at seven days (p = 0.9239).

Time to resume work was 4.50 ± 1.09 days in Group A and 4.64 ± 1.22 days in Group B (p = 0.7116). Mean patient satisfaction scores were also comparable (5.44 ± 1.09 vs. 5.36 ± 1.08; p = 0.8242) (Table 2).

**Table 2:** Different parameters in the study population Data were presented as mean and standard deviation (SD). Unpaired t-tests were used to compare variables. p < 0.05 was considered statistically significant. ASA: American Society of Anesthesiologists, BMI: body mass index, VAS: visual analogue scale.

Parameter	Group A (day-care, n = 16)	Group B (short-stay, n = 25)	p-value
Mean age (years)	33.75 ± 11.21	39.32 ± 10.58	0.1163
Mean BMI (kg/m²)	23.87 ± 2.55	24.36 ± 3.23	0.6157
Mean ASA Grade	1.00 ± 0.00	1.04 ± 0.20	0.4307
Mean hernia size (cm)	2.69 ± 0.48	1.84 ± 0.98	0.0028
Operative duration (minutes)	66.38 ± 4.60	66.56 ± 4.06	0.8933
VAS pain score (6 hours)	2.75 ± 0.86	3.04 ± 0.84	0.2913
VAS pain score (7 days)	1.19 ± 0.40	1.20 ± 0.41	0.9239
Time to resume work (days)	4.50 ± 1.09	4.64 ± 1.22	0.7116
Patient satisfaction score	5.44 ± 1.09	5.36 ± 1.08	0.8242

Complication rates were minimal. Vascular injury occurred in only one (4.0%) patient in Group B, and none were reported in Group A (p = 0.4179, chi-square value: 0.6560, df = 1). Urinary retention was seen in two patients (8.0%) in Group B, with no cases reported in Group A (p = 0.2460, chi-square value: 1.3456, df = 1). No cases of port-site infection, seroma, scrotal edema, or recurrence were observed in either group. Overall postoperative recovery was uneventful in all patients. 

## Discussion

Inguinal hernia repair is one of the most frequently performed general surgical procedures worldwide, with laparoscopic TEP repair increasingly favored due to its reduced postoperative pain, earlier recovery, and lower incidence of chronic groin discomfort compared to open mesh repair. With advances in anesthesia, perioperative care, and minimally invasive techniques, day-care surgery and short-stay surgery have emerged as cost-effective and patient-centered approaches. The present study compared the safety and acceptability between day-care surgery versus short-stay surgery in patients undergoing laparoscopic TEP hernia repair, focusing on operative outcomes, complications, recovery parameters, and patient satisfaction.

The mean age of patients in Group B (39.32 ± 10.58 years) was higher than in Group A (33.75 ± 11.22 years), though the difference was not statistically significant (p = 0.1163). This finding is consistent with observations by Burcharth et al., who reported that the incidence of inguinal hernia repair increases with age, particularly in males over 30 years [11].

All patients in our series were male, with Group B comprising 25 (61.0%) and Group A 16 patients (39.0%). This male predominance aligns with the epidemiological trends described by Srivastava et al., who emphasized the substantially higher prevalence of inguinal hernia among men due to anatomical differences, such as the structure of the inguinal canal and the presence of the spermatic cord [3]. Lifestyle factors and occupations involving heavy lifting further increase the risk in men, underscoring the importance of gender-specific epidemiological understanding in early detection and prevention [3].

Right-sided inguinal hernia was the most common diagnosis in both groups (15 (93.8%) in Group A and 23 (92.0%) in Group B), with no statistically significant difference (p = 0.8337, chi-square value: 0.0441, df = 1). This pattern reflects global epidemiological observations. Gavriilidis et al. attributed this predominance to delayed atrophy of the processus vaginalis following slower descent of the right testis, highlighting the anatomical predisposition to right-sided hernia formation [1].

We observed that the mean duration of surgery was comparable in both groups (66.38 ± 4.60 minutes in Group A and 66.56 ± 4.06 minutes in Group B), with no statistically significant difference (p = 0.8933). Myers et al. have reported that operative time in laparoscopic repair varies with hernia size, laterality, and surgeon experience [2], whereas Bittner et al. highlighted that with increasing proficiency, the duration of TEP repair stabilizes around 60-75 minutes, aligning well with our findings [12].

Vascular injury occurred in only one patient (4.0%) in Group B and none in Group A, with no statistical significance (p = 0.4179, chi-square value: 0.6560, df = 1). Belyansky et al. highlighted the risk of inferior epigastric vessel injury during TEP [13], while Köckerling et al. emphasized that vascular complications are more likely in inexperienced hands or complex cases [14]. Our findings fall within the reported range of 0.1%-4%.

Pain management was effective in both groups, with mean VAS scores at six hours of 2.75 ± 0.86 in Group A and 3.04 ± 0.84 in Group B (p = 0.2913). At seven days, the scores were 1.19 ± 0.40 and 1.20 ± 0.41, respectively (p = 0.9239). These findings demonstrate the benefits of multimodal analgesia, which, as Kim et al. reported, reduces opioid consumption and associated side effects while improving comfort and recovery [15]. Yang and Tung similarly noted that multimodal regimens minimize the need for additional analgesics, consistent with our findings of reduced analgesic interventions [16].

The incidence of postoperative nausea and vomiting (PONV) was minimal in our series, reflecting effective anesthetic protocols and early hydration strategies. Chauhan and Chheda emphasized that successful PONV management is vital for patient comfort and discharge readiness, especially in ambulatory surgery [17].

No cases of port-site infection or scrotal edema were observed in either group, underscoring the importance of aseptic techniques and careful postoperative wound care. Gavriilidis et al. confirmed that strict adherence to sterile protocols markedly decreases infection risk [1]. Similarly, no cases of seroma were reported in our study. Chowbey et al. noted that seroma formation, a known complication of laparoscopic repair, can be minimized by meticulous dissection and sac management techniques, which were consistently applied in our series [18].

Urinary retention was reported in two patients (8.0%) in Group B but none in Group A, though this difference was not significant (p = 0.2460, chi-square value: 1.3456, df = 1). Lin et al. reported that urinary retention is influenced by factors such as male sex, anesthesia type, fluid administration, and opioids [19]. Preventive measures including preoperative voiding, limited intraoperative fluids, and early ambulation likely contributed to the low incidence in our study.

Chronic groin pain was not reported in any patient during follow-up. Köckerling et al. demonstrated that laparoscopic approaches, particularly TEP, reduce the risk of chronic pain through minimal fixation and avoidance of nerve injury, supporting our findings [14].

No hernia recurrence was observed in either group during follow-up. Yang and Tung reported recurrence rates as low as 1%-2% with TEP when performed by experienced surgeons [16]. Our recurrence-free outcome reaffirms the importance of complete dissection, adequate mesh coverage, and proper fixation.

Patient satisfaction was high in both groups, with mean scores of 5.44 ± 1.09 in Group A and 5.36 ± 1.08 in Group B (p = 0.8242). These results are similar with the findings of Duwal and Manandhar [20], Rathod et al. [21], and Mir et al. [22], who all reported consistently high satisfaction following laparoscopic repair, often related to faster recovery, minimal scarring, and less postoperative discomfort. Ugraiah et al. reported that early ambulation was associated to improved satisfaction [23].

From a health-economic perspective, day-care surgery offered clear advantages over short-stay management. Our findings are supported by Chowbey et al., who documented a 20%-30% reduction in hospital costs with day-care models [18]. Kim et al. [15] and Wakasugi et al. [24] also emphasized the safety and cost-effectiveness of day-care TEP in appropriately selected patients.

The success of day-care surgery in our cohort was further enhanced by thorough preoperative education, which ensured compliance, reduced anxiety, and minimized readmissions. Chauhan and Chheda highlighted the pivotal role of structured education in ambulatory settings, a finding echoed by our experience [17].

Follow-up at seven days and at three months was integral to monitoring complications and ensuring long-term success. Lin et al. emphasized that continuity of care, including telephonic support, is crucial for safe early discharge [19].

Finally, mesh selection and anaesthetic strategy played a vital role in outcomes. We used polypropylene mesh with large pores, which is associated with lower risks of chronic pain and infection. General anesthesia was administered in all cases, providing optimal surgical conditions, and short-acting agents minimized postoperative side effects. Kim et al. reinforced the importance of anesthesia protocols tailored for day-care surgery to enhance recovery and discharge readiness [15].

Overall, our findings affirm that day-care laparoscopic TEP inguinal hernia repair is a safe, effective, and economically viable option, with outcomes comparable to short-stay surgery while offering distinct advantages in terms of cost and recovery.

Limitations

The sample size was relatively small, with only 41 cases, which may not be sufficient to draw robust conclusions. Being a single-center study conducted in a tertiary care hospital, the findings may be influenced by institutional practices, and hospital bias cannot be entirely excluded.

## Conclusions

The present study evaluated the safety, efficacy, and acceptability of day-care versus short-stay surgery in patients undergoing laparoscopic TEP inguinal hernia repair. Both groups were comparable with respect to age, BMI, ASA Grade, and operative duration, with no statistically significant differences observed. PONV and pain were generally mild and similar between the groups. Complications were uncommon and comparable, limited to two cases of urinary retention and one vascular injury in the short-stay group, while seroma, scrotal edema, port-site infection, and hernia recurrence occurred rarely in both groups. Importantly, the day-care group achieved these comparable outcomes despite having significantly larger hernias at baseline, demonstrating the feasibility and safety of the day-care approach even in more challenging cases. These findings indicate that day-care TEP repair is as safe, effective, and acceptable as short-stay surgery, supporting its use as a viable option for appropriately selected patients while optimizing hospital resource utilization.
